# The Role of Pulmonary Surfactants in the Treatment of Acute Respiratory Distress Syndrome in COVID-19

**DOI:** 10.3389/fphar.2021.698905

**Published:** 2021-06-29

**Authors:** Shengguang Wang, Zhen Li, Xinyu Wang, Shiming Zhang, Peng Gao, Zuorong Shi

**Affiliations:** ^1^ School of Pharmacy, Shandong University of Traditional Chinese Medicine, Jinan, China; ^2^ School of Chinese Medicine, Shandong University of Traditional Chinese Medicine, Jinan, China

**Keywords:** pulmonary surfactant, COVID-19, SARS-cov-2, ARDS, NRDS

## Abstract

Lung alveolar type-II (AT-II) cells produce pulmonary surfactant (PS), consisting of proteins and lipids. The lipids in PS are primarily responsible for reducing the air-fluid surface tension inside the alveoli of the lungs and to prevent atelectasis. The proteins are of two types: hydrophilic and hydrophobic. Hydrophilic surfactants are primarily responsible for opsonisation, thereby protecting the lungs from microbial and environmental contaminants. Hydrophobic surfactants are primarily responsible for respiratory function. Severe acute respiratory syndrome coronavirus-2 (SARS-CoV-2) enters the lungs through ACE-2 receptors on lungs and replicates in AT-II cells leading to the etiology of Coronavirus disease – 2019 (COVID-19). The SARS-CoV-2 virus damages the AT-II cells and results in decreased production of PS. The clinical symptoms of acute respiratory distress syndrome (ARDS) in COVID-19 patients are like those of neonatal respiratory distress syndrome (NRDS). The PS treatment is first-line treatment option for NRDS and found to be well tolerated in ARDS patients with inconclusive efficacy. Over the past 70°years, a lot of research is underway to produce natural/synthetic PS and developing systems for delivering PS directly to the lungs, in addition to finding the association between PS levels and respiratory illnesses. In the present COVID-19 pandemic situation, the scientific community all over the world is searching for the effective therapeutic options to improve the clinical outcomes. With a strong scientific and evidence-based background on role of PS in lung homeostasis and infection, few clinical trials were initiated to evaluate the functions of PS in COVID-19. Here, we connect the data on PS with reference to pulmonary physiology and infection with its possible therapeutic benefit in COVID-19 patients.

## Introduction

Human pulmonary surfactant (PS) is an endogenous lipoprotein complex produced naturally in the lungs. PS forms a layer on the alveolar epithelium and is responsible in reducing surface tension at the air-fluid interface on the alveolar surface (Agassandian and Mallampalli, 2013). The reduced alveolar surface tension will allow the expansion of alveoli and allows gas exchange (Seadler et al., 2020). Human PS contains phospholipids, mainly dipalmitoylphosphatidylcholine (DPPC), and surfactant proteins- A, B, C, and D. The PS is present as a barrier when inhaled particle and noxious agents come in contact with it and enhances the clearance of particles. PS also participate in host defense against infections and inflammation. The loss or deficiency in endogenous surfactant is implicated with respiratory disorders (Wert et al., 2009). During the gestation period, the production of endogenous lung surfactant results in lowering alveolar surface tension and stabilizes the alveoli to prevent the lung from collapsing at resting transpulmonary pressures. Premature infants are highly likely to be PS deficient, which causes increased surface tension leading to lung collapse and results in neonatal respiratory distress syndrome (NRDS). NRDS is associated with fast breathing, increased heart rate and apoxia, which in certain cases may lead to death (Khawar and Marwaha, 2021). PS therapy is currently the first-line treatment for NRDS.

During the COVID-19 pandemic, patients admitted in intensive care units are mainly those with clinical symptoms of acute respiratory distress syndrome (ARDS). The severe acute respiratory syndrome coronavirus (SARS-CoV)-2-induced lung injury in COVID-19 patients may lead to respiratory failure. Emerging evidence on respiratory mechanisms suggests that clinical symptoms of ARDS in COVID-19 patients resemble to those of NRDS caused by surfactant deficiency.

In this review, we connect the current understanding of the pathophysiology of lungs in COVID-19 patients with the possible role of PS in circumventing ARDS symptoms in COVID-19 patients.

## Pulmonary Surfactant in Lung Homeostasis

**FIGURE 1:**
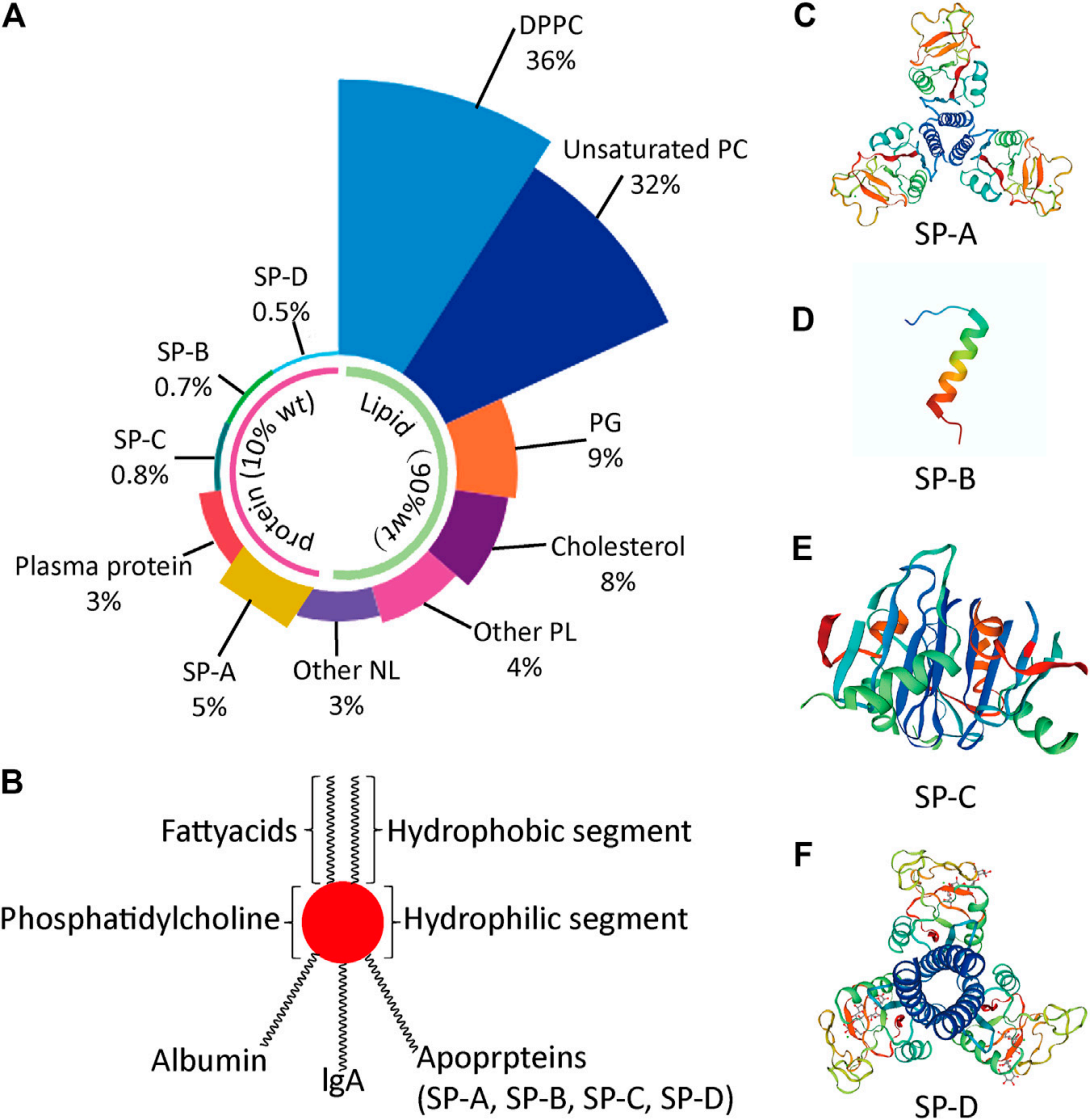
Lung pulmonary surfactant (PS) **(A)** Relative precent of various components in PS **(B)** Representative structure of PS **(C)** Molecular representation of surfactant protein (SP)-A **(D)** Molecular representation of SP-B **(E)** Molecular representation of SP-C **(F)** Molecular representation of SP-D. DPPC, dipalmitoyl phosphatidylcholine; NL, neutral lipid; PC, phosphatidylcholine; PG, phosphatidylglycerol; PL, phospholipid; SP, surfactant protein.

PS is an important biosurfactant in human. PS lines the alveoli and terminal bronchioles, thereby protecting the lungs from atelectasis (Han and Mallampalli, 2015). PS is synthesized in type II alveolar epithelial cells, stored in lamellar bodies, and is secreted via exocytosis into the alveolar lumen. PS is a complex mixture comprising 90% lipids and 10% surfactant proteins by weight. The lipids are made of DPPC (36%), unsaturated phosphatidylcholine (PC; 32%), phosphatidylglycerol (PG; 8%), cholesterol (7%), other phospholipids (PL; 4%) and other neutral lipids (NL; 3%). The surfactant proteins consist of plasma protein (3%), surfactant protein (SP)-A (5%), SP-B (0.7%), SP-C (0.8%) and SP-D (0.5%) as depicted in Figure 1A) (Bernhard, 2016).

The PS lipids forms a monolayer at the air-fluid interface, reduces the surface tension to a minimum of <10 mN/m and thus, prevents the collapse of alveoli and maintains the alveolar stability (Sunde et al., 2017). The main active component of PS lipids is DPPC.

The SPs are classified into hydrophilic SPs (SP-A and SP-D) and hydrophobic SPs (SP-B and SP-C; Figures 1C–F). The details of SPs and PS lipids are provided in Table 1 (Wang et al., 2020). SP-A and SP-D are highly ordered collagen-like oligomeric glycoprotein belonging to collectin family. These two SPs are part of innate immune response and protect the lungs against inhaled chemicals and microorganisms via stimulating phagocytosis by alveolar macrophages. They are also involved in surfactant metabolism. SP-A is the most abundant SP and accounts for 2–3% w/w of total SPs. It does not have any effect on surface tension at air-fluid interface in alveoli. However, it enhances the phospholipid absorption process to the air-fluid interface, regulates the PS secretion by AT-II cells, binds specific carbohydrate moieties found on lipids and on the surface of microorganisms and prevents the inhibition of surfactant function by plasma proteins which are leaked into the alveolar space (Echaide et al., 2017). The encoded protein may also be involved in surfactant metabolism. SP-B and SP-C are apolipoproteins comprising of 1–2% w/w of total SPs (Echaide et al., 2017). These are involved in the spreading of the surfactant layer at the air-fluid interface and thus reduce the surface tension (Weaver and Conkright, 2001). The SP-B enhances the rate of spreading and increases the stability of monolayers.

**TABLE 1:** Characteristics and details of pulmonary surfactant proteins and surfactant lipids.

Name	Size	Chemistry	Major functions	Ref
SP-A	28–36 kDa	Hydrophilic	Involved in facilitating phagocytosis, inhibition of phospholipase A_2_ activity and maintaining surfactant integrity during lung injury	[1]
		Octadecameric glycoprotein, acidic		
SP-B	8 kDa	Hydrophobic.	Involved in decreasing the surface tension and enhancing adsorption of PL at air-water interface. Deficiency results in severe respiratory failure	[2]
		Disulfide linked homodimer with 79 amino acids (AA)		
SP-C	4.2 kDa	Hydrophobic.	Involved in stabilizing phospholipids, increasing the viscosity of air-water interfacial film	[2]
		α-helical protein with 35 AA	Deficiency results in minimal effect on respiratory function	
SP-D	43 kDa	Hydrophilic. Dodecameric glycoprotein with 4 trimmers	Involved in regulating surfactant metabolism and promotes phagocytosis by alveolar cells	[3]
1,2-Dipalmitoyl-sn-glycero-3-phosphatidylcholine	734.05 gmol^-1^	PC16:0/16:0, C_40_H_80_NO_8_P	Involved in the generation of near-zero surface tension	[3]
1-Palmitoyl-2-oleoyl-sn-glycero-3-phosphocholine (POPC)	760.09 gmol^-1^	PC 16:0/18:1	Involved in making the membrane fluid at body temperature	[3,4]
		C_42_H_82_NO_8_P		
1-Palmitoyl-2-palmitoleoyl-sn-glycero-3-phosphocholine (PPPC)	732.04 gmol^-1^	PC 16:0/16:1, C_40_H_78_NO_8_P	Involved in regulating respiratory rate and surface dynamics of surfactant	[3,4]
1-Palmitoyl-2-myristoyl-sn-glycero-3-phosphocholine	706 gmol^-1^	PC16:0/14:0, C_38_H_76_NO_8_P	Involved in regulating respiratory rate and alveolar macrophages function to improve protection	[4,5]
1,2-Dipalmitoyl- sn-glycero-3-phosphoglycerol (DPPG)	722.98 gmol^-1^	C_38_H_75_O_10_P	Involved in reducing permeability of benzo [*a*]pyrene	[4,5]
1-Palmitoyl-2-oleoyl-sn-glycero-3-phosphoglycerol (POPG)	749.02 gmol^-1^	C_40_H_77_O_10_P	The most abundant PG in human *p*S. Enhances fluidization of film, inhibits macrophage proinflammatory responses and antiviral	[4,5]
Phosphatidylserine	792.09 gmol^-1^	C_42_H_82_NO_10_P	Involved in determining the cellular and subcellular distribution of quinidine	[2,4]
PE	299.22 gmol^-1^	C_9_H_18_NO_8_P	Involved in stabilizing membrane protein by initiation of lateral pressure and curvature stress	[2,4]
Phosphatidylinositol	334.21 gmol^-1^	C_9_H_19_O_11_P	Involved in increasing the rate of alveolar fluid clearance and stabilization of surfactant mono layer	[4,6]
Cholesterol	386.66 gmol^-1^	C_17_H_46_O	Involved in increasing the surfactant fluidity	[4,7]

Note: AA, amino acid; DPPC, dipalmityl phophotidylcholine; PE, phosphatidylethanolamine; PS, Pulmonary surfactant; SP, surfactant protein; C, Carbon; H, Hydrogen: O, Oxygen; *p*, Phosphorus; N, Nitrogen.

References.

[1] J.A. Whitsett, The molecular era of surfactant biology, Neonatology. 105 (2014) 337–343. doi:10.1159/000360649.

[2] F.P.S. Yu, D. Islam, J. Sikora, S. Dworski, J. Gurka, L. López-Vásquez, M. Liu, W.M. Kuebler, T. Levade, H. Zhang, J.A. Medin, Chronic lung injury and impaired pulmonary function in a mouse model of acid ceramidase deficiency., Am. J. Physiol. Lung Cell. Mol. Physiol. 314 (2018) L406–L420. doi:10.1152/ajplung.00223.2017.

[3] F. Wang, J. Liu, H. Zeng, Interactions of particulate matter and pulmonary surfactant: Implications for human health., Adv. Colloid Interface Sci. 284 (2020) 102,244. doi:10.1016/j.cis. 2020.102,244.

[4] S.E. Wert, J.A. Whitsett, L.M. Nogee, Genetic disorders of surfactant dysfunction, Pediatr. Dev. Pathol. 12 (2009) 253–274. doi:10.2350/09–01-0,586.1.

[5] U. Klenz, M. Saleem, M.C. Meyer, H.J. Galla, Influence of lipid saturation grade and headgroup charge: A refined lung surfactant adsorption model, Biophys. J. 95 (2008) 699–709. doi:10.1529/biophysj.108.131,102.

[6] D.R. Voelker, M. Numata, Phospholipid regulation of innate immunity and respiratory viral infection., J. Biol. Chem. 294 (2019) 4,282–4,289. doi:10.1074/jbc.AW118.003229.

[7] A. Kelly, C. McCarthy, Pulmonary Alveolar Proteinosis Syndrome., Semin. Respir. Crit. Care Med. 41 (2020) 288–298. doi:10.1055/s-0039–3402,727.

## Pulmonary Surfactant Deficiency/Dysfunction

In anticipation of birth during gestation period, the alveoli start producing PS in 24th week and reaches to the peak production in 34th week. The endogenous cortisol stimulates the production of PS during gestation. Premature infants, especially those born before 34th weeks, have immature lungs and are deficient in *p*S. These infants have difficulty in breathing and develop a condition called NRDS (Nogee, 2019). Pregnant women who are at the risk of premature delivery are given betamethasone for 48 h before delivery to improve the lung maturity and reduce the risk of developing NRDS. The genetic disorders comprising the mutations in SP-B and SP-C are reported to cause surfactant dysfunction, leading to the development of NRDS. Observational studies suggested that humans with ARDS have altered PS composition and its functions. AT-II epithelial cells are reported to be the primary site of influenza virus replication. Mice infected with influenza virus have shown lower amounts of phosphatidylcholine and alters the metabolism of PS, which are attributed to the development of ARDS (Woods et al., 2016).

The effects of SP deficiencies or dysfunctions is paramount in the pathogenesis of neonatal respiratory diseases (Verlato et al., 2018). In neonates, SP-A is critical in lung immune system while SP-B is important in sustaining respiratory physiology. It was also substantiated that there is a significant lack of surfactant protein found in preterm newborns with RDS or had experienced failure in extubation than that of newborns with normal functioning lungs (Ballard et al., 2019). It is also known that polymorphisms of SP-A, B and D showed association with idiopathic pulmonary fibrosis and various other pulmonary diseases. Chang et al. reported that SP-A +186A/G and SP-B 1580C/T polymorphisms results in the elevated risk of preterm NRDS; on the other hand, polymorphisms of SP-B –18A/C, SP-D Met11 ThrT/C, and Ala160 ThrG/A genes are not associated to the risk of NRDS (Chang et al., 2016).

The beneficial use of surfactant protein as a treatment in neonates with RDS has been a breakthrough and has been studied in-depth for neonatal medicine in the past 3 decades (Speer et al., 2013). Thus, it is logical to hypothesize that restoration of PS does improve the lung function (Echaide et al., 2017) and circumvent the symptoms of NRDS in infants and ARDS in adults.

## Applications of Pulmonary Surfactant

The primary application of pulmonary surfactants is in the treatment of NRDS in premature infants. However, the studies did not demonstrate significant benefit of pulmonary surfactants in ARDS. Meta-analysis of randomized controlled trials for the effect of surfactant in adult patients with ARDS (Ballard et al., 2019) revealed neither improvements in the mortality nor improvement in oxygenation. Marcel Filoche et al. proposed that insufficient delivery of PS to the lungs in adults could be the reason for showing the efficacy in adults (Speer et al., 2013). One of the postulations put forward to explain the observed differences in clinical efficacy of PS in NRDS and ARDS is that in case of NRDS, the surfactants are administered well in advance before the RDS becomes severe in infants who are at the risk of developing NRDS. Thus, it is worth to explore the option of checking the efficacy of PS in early stages of ARDS. However, this approach requires the identification of patients who are at the stage of developing ARDS. Thus, identification of PS levels in serum would predict the occurrence of ARDS.

The clinical efficacy of PS is also being actively investigated in other pulmonary diseases such as asthma and pneumonia (Choi et al., 2020). One study reported that PS improved lung function in an acute asthma exacerbation but not in stable asthma (Tepper et al., 2012). One study reported the administration of PS improved oxygenation in Gram-negative lobar pneumonia and in HIV-infected patients with P. carinii pneumonia or RSV pneumonia (Han and Mallampalli, 2015). Another study reported that PS improved the pulmonary function in adult patient with stable chronic bronchitis (Agudelo et al., 2020). In addition, PS is reported to decrease the cytokine release, synthesis of inflammatory mediators, lymphocyte proliferation, immunoglobulin production, and expression of adhesion molecules. Another study reported PS improves the anti-inflammatory effect of amikacin. All the above observations suggest the possible role of surfactants in modulating the immune responses in pulmonary diseases.

The SP-A and SP-D are reported to bind to viruses (influenza A, human immunodeficiency virus (HIV), respiratory syncytial virus (RSV), SARS-CoV) and inhibit their activity of the viruses through viral neutralization, agglutination, and enhanced phagocytosis (Cañadas et al., 2020).

## Therapeutic Pulmonary Surfactants

There are two types of therapeutic PS: natural and synthetic. Natural PS are derived from animals while synthetic PS contain peptides that mimic SP-B and SP-C. Therapeutic PS are the first-line treatment option for NRDS (Whitsett, 2014; Hentschel et al., 2020). The natural therapeutic PS are being sourced from bovine, porcine, and human amniotic fluid. Currently the use of human amniotic fluid for sourcing therapeutic PS are halted mainly because of non-availability and cost. The advantage of natural surfactants is that they contain surfactant-associated proteins and thus results in better spreading and lung defense properties.

Due to the difficulties in sourcing animal derived surfactant, well-defined synthetic surfactants were developed. Initially, the synthesis of artificial SP-B and SP-C used for the treatment of neonatal RDS was indeed challenging. It was also reported that synthetic surfactant containing only one protein has not found success (Johansson and Curstedt, 2019). Both synthetic and natural surfactants are found to be effective in RDS with natural surfactants containing SP-B and SP-C are found to be superior in clinical efficacy.

To date, the list of natural surfactants, and synthetic surfactants developed for the treatment of respiratory infections are shown in Table 2. First generation synthetic surfactants were prepared in combination of DPPC with either egg phosphatidylglycerol (ALEC^®^) or hexadecanol and tyloxapol (Exosurf^®^) (Zhang et al., 2011). However, the first-generation surfactants do not contain either SP-B or SP-C peptide mimics, thus limiting their clinical efficacy. The second-generation of synthetic surfactants contain either SP-B (Surfaxin^®^) or SP-C (Venticute^®^) peptide (Bae et al., 2016). The second-generation of synthetic surfactants are found to be clinically effective, suggesting the presence of SP-B and SP-C in surfactants are essential.

**TABLE 2:** Clinical trials on pulmonary surfactants for the treatment of ARDS in COVID-19 patients.

Surfactant	Dose	Administration route	Study type	Primary purpose	NCT number
Poractant alfa	50 mg/kg only once	Bronchial fibroscopy	Interventional	Treatment using Curosurf^®^ in adult acute respiratory distress syndrome due to COVID-19	NCT04384731
Poractant alfa	30 mg/kg once a day for 3 days	Endotracheal intubation	Interventional	Treatment using poractant alfa - curosurf for SARS-cov-19 ARDS (Covid-19)	NCT04502433
Bovine lung extract surfactant	50 mg/kg once a day for 3 days	Endotracheal intubation	Interventional	Treatment using London’s exogenous surfactant study for COVID-19 (LESSCOVID)	NCT04375735
Bovine lung extract surfactant	150 mg twice a day for 5 days	Inhalation	Observational	Treatment using Surfactant-BL in adult ARDS due to COVID-19	NCT04568018
Lucinactant	80 mg	Injection	Interventional	Treatment by assessing the safety and preliminary tolerability of lyophilized lucinactant in adults with Covid-19	NCT04389671
COVSurf	N/A	N/A	Interventional	Treatment using delivery of the surfactant to the lungs	NCT04362059
Exogenous surfactant		Inhalation	Interventional	Evaluation of the effect of exogenous surfactant through nebulizer mask on clinical outcomes in Covid-19 patients	NCT04847375
Biological: AT-100 (rhSP-D)	75 or 150 mg once a day for 7 days	Intratracheal administration	Interventional	Treatment: Safety study on AT-100 in treating adults with severe COVID-19 infection	NCT04659122

Note: N/A, Not applicable; ARDS, Acute Respiratory Distress Syndrome; NCT, National Clinical Trials.

Colfosceril palmitate is a first generation commercially available artificial surfactant (Law et al., 2014; Sardesai et al., 2017). At present, it is under the state of cancellation in the post-marketing stage because of adverse effects. In addition to being useful in RDS, it has also shown to significantly reduce the risk of pneumothoraces, pulmonary interstitial emphysema and mortality, bronchopulmonary dysplasia, intraventricular hemorrhage and patent ductus arteriosus. Sinapultide, also known as KL4 peptide, mimics human SP-B. It is administered as its aqueous dispersion with the phospholipids. Lucinactant is a synthetic surfactant containing sinapultide, and lipids, DPPC, palmitoyloleoyl phosphatidylglycerol (POPG) and a palmitic acid. Pumactant is another synthetic surfactant containing naturally occurring phospholipids DPPC and PG.

Calfactant is a natural pulmonary surfactant from calf lungs containing phosphatidylcholine, SP-B and SP-C. Beractant is another natural pulmonary surfactant from bovine lungs containing phosphotidylcholine, triglycerides, fatty acids, SP-B and SP-C. Portactant alfa is another natural pulmonary surfactant from porcine lungs containing phosphatidylcholine, dipaImitoylphosphatidylcholine, SP-B and SP-C.

## Pulmonary Surfactants in COVID-19

**FIGURE 2:**
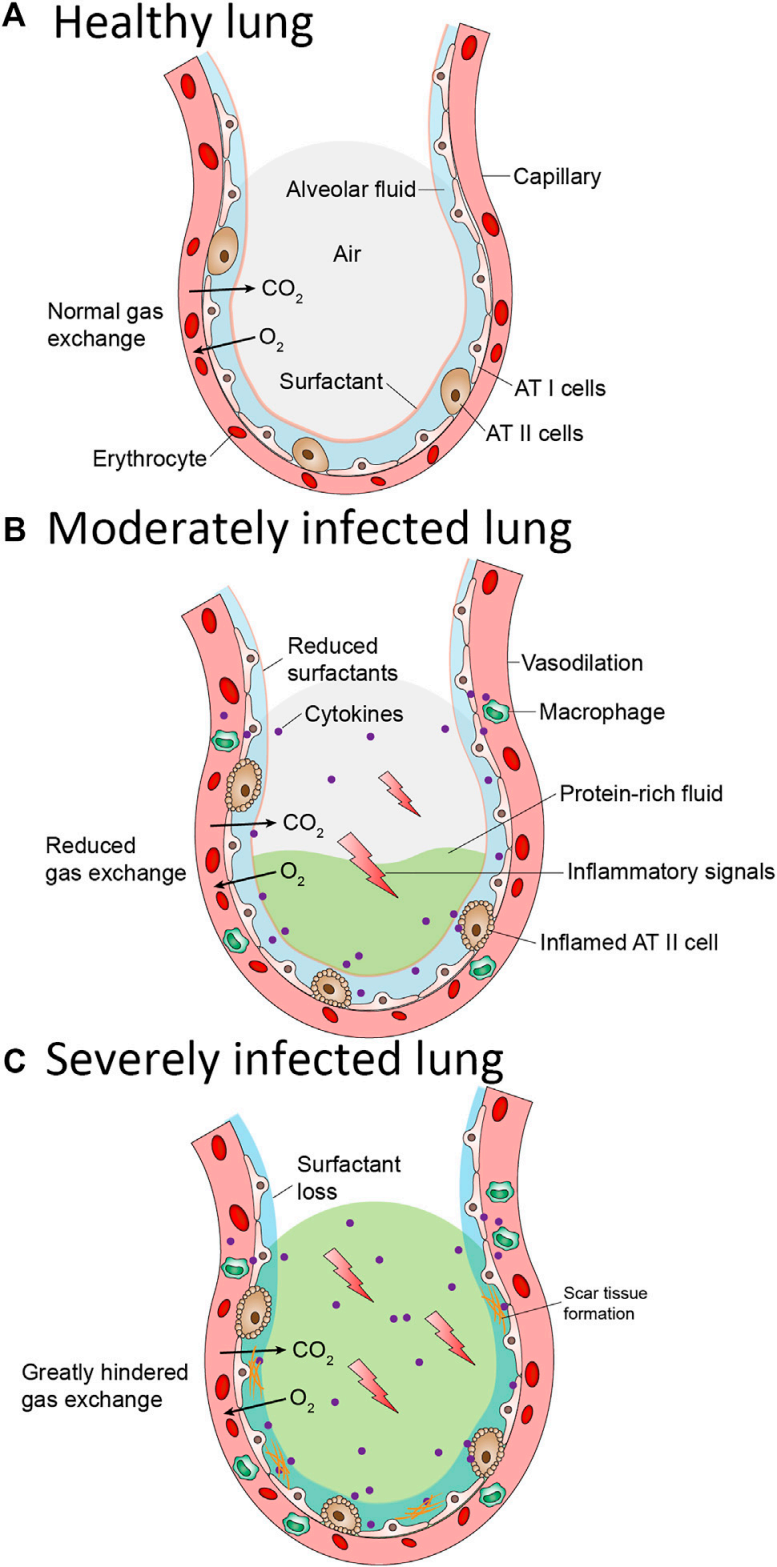
Pathological changes in the lung alveolus during COVID-19 **(A)** Normal alveolus is wrapped with capillaries containing red blood cells. Oxygen in the alveolus is exchanged with carbon dioxide in the capillaries. The alveolus surface contains alveolar Type I and Type II cells. Type I cells enables gas exchange. Type II cells secrete pulmonary surfactant (PS). PS lines the alveolus and prevent it from collapsing **(B)** In a moderately infected lung, Alveolar Type II cells are inflamed resulting in reduced pulmonary surfactant. Surface tension and pressure increase inside the alveolus affecting the gas exchange. Vasodilation of the capillary occurs resulting in the release of inflammatory cytokines and accumulation of protein-rich fluid inside the alveolus **(C)** In severely infected lung, the alveolar type II cells become more inflamed thereby resulting in complete loss of pulmonary surfactant. Scar tissue on the alveolar surface began to form. The release of inflammatory cytokines is increased, and more protein-rich fluid accumulate inside the alveolus. The oxygen/carbon dioxide exchange is greatly hindered and thus patients in this stage must undergo intubation as an aid to breathe.

SARS-CoV-2 enters the body through lungs via binding of viral spike protein with angiotensin converting enzyme 2 (ACE-2) receptor (Mason, 2020). After entry, SARS-CoV-2 is postulated to destroy type II alveolar cells, the site for the synthesis of pulmonary surfactants, resulting in decreased production of *p*S. Decreased surfactant production causes atelectasis and reduced the pulmonary compliance. The patients with Coronavirus disease 2019 (COVID-19) are presented with clinical symptoms which are very similar to those observed in NRDS (Nieman et al., 2018; Schousboe et al., 2020) for which deficiency in PS is the primary cause (Figure 2). Decreased concentration of PS, altered composition of PS and mutations in PS are reported to be the critical factors in COVID-19 mortality (Weiskirchen, 2020). Mirastschijski et al. has suggested to include pulmonary surfactants therapy in the early stages together with standard ARDS care. Preliminary observations from lung autopsies of COVID-19 patients found that pulmonary surfactant increased blood oxygenation, reduced pulmonary edema, and ameliorated the excessive inflammatory reaction (Mirastschijski et al., 2020). In addition, PS is reported to have the ability to recognize the SARS-CoV-2 spike protein and thereby activate the macrophages for phagocytosis (Matera et al., 2020). This evidence motivated the interventional clinical trials to investigate the clinical effectiveness of PS in COVID-19 patients.

Gattinoni et al. (Gattinoni et al., 2020) has classified COVID-19 patients into two different groups; one group develops acute respiratory distress syndrome (ARDS) with low compliance and another group develops non-ARDS with normal compliance. Gene expression studies on lung biopsy cells in COVID-19 patients have confirmed the downregulation of pulmonary surfactant proteins and their metabolism which has provided a scientific base to advocate further studies on investigating the usefulness of surfactant therapy in COVID-19 patients (Islam and Khan, 2020). Clinical trials are underway to determine the association of SP-D levels (NCT04618861) and SP genetic variants (NCT04650191) with severity of COVID-19 infection (Surfactant Protein D Levels in Covid-19 Infection: Case-Control Study; Surfactant Protein Genetic Variants in COVID-19 Infection) and the results are not yet available.

Peter et al. (Schousboe et al., 2020) has postulated that COVID-19 patients with pulmonary surfactant deficiency develop symptoms resembling neonatal respiratory distress syndrome (NRDS). A clinical trial (NCT04609488) is underway to determine the levels of surfactant proteins in COVID-19 patients to delineate the association between surfactant deficiency and progression of COVID-19 disease.

Lung surfactant therapy is a standard, safe and effective therapy for the treatment of ARDS in neonates, however clinical trials on recombinant SP-C based surfactant was found to be ineffective in the treatment of ARDS in adults (Spragg et al., 2004). The natural surfactants, compared to synthetic surfactants, are reported to be superior in improving the blood oxygenation and shortening the ventilation time in infants (Ainsworth et al., 2000; Been and Zimmermann, 2007). These observations suggest that early administration of natural surfactant to COVID-19 patients might be beneficial to improve the pulmonary function (Mirastschijski et al., 2020).

A recent review article by Francesco et al. (Cattel et al., 2021) has highlighted the potential use of exogenous surfactants early in the treatment of COVID-19 ARDS. Kumar et al. (Kumar, 2020) has proposed an innovative hypothesis that co-aerosolized exogenous pulmonary surfactant and ambroxol can be a potential therapeutic option for the treatment of COVID-19 ARDS. The hypothesis was made based on reported evidences on beneficial effects of exogenous surfactants (Davidson et al., 2006; Dushianthan et al., 2012; Zhang et al., 2013; Meng et al., 2019) and ambroxol (Malerba and Ragnoli, 2008; Paleari et al., 2011; Kantar et al., 2020) in the treatment of ARDS. However, this hypothesis is yet to be tested. A prospective observational cohort study revealed that autoimmunity in severe COVID-19 patients is mediated through binding of immunoglobulin A (IgA) antibodies to human surfactant protein B (SP-B) and surfactant protein C (SP-C) leading to reduced levels of pulmonary surfactant (Sinnberg et al., 2021).

Abbas et al. (Abbasi et al., 2021) has made a serendipitous observation that non-invasive ventilation improved the survival of mice with bacterial pneumonia and the improved survival is associated with the expression of surfactant protein A. Majority of the COVID-19 patients are also reported to be co-infect with other pathogens (Hughes et al., 2020; Jiang et al., 2020; Kim et al., 2020; Roh et al., 2021) and many research papers (Floros and Phelps, 2020; Tekos et al., 2020; Xu et al., 2020) have highlighted that SP-A variants have shown beneficial effects in the treatment of ARDS in COVID-19 patients under different scenarios. Thus, in an opinion article by Abbs et al. (Abbasi et al., 2021), the team has expressed that non-invasive ventilation using high-flow nasal cannula (HFNC) may be beneficial for COVID-19 patients which warrants further laboratory and clinical studies to confirm (Abbasi et al., 2021).

A clinical study (Choreño-Parra et al., 2021) has concluded that serum SP-D level is high only in pandemic influenza A (H1N1) but not in COVID-19. However, contradicting to this study, another clinical study (Kerget et al., 2020) has concluded that human SP-D levels is higher in individuals with COVID-19 compared to those without COVID-19. The contradictory results from these two studies may be due to differences in the population demographics, and objectives of the study. Thus, more clinical studies are warranted to confirm the association between SP-D levels and progression of COVID-19 infection.

In a case study of a 48-year-old-male non-smoker COVID-19 patient with comorbidities of hyperlipidemia and prediabetes (Heching et al., 2021), it is reported that administration of surfactant (Calfactant) directly to the lungs has improved oxygenation. This observation rises a hope that surfactant therapy would be beneficial for treating ARDS in COVID-19 and thus warrant further detailed investigations to confirm the therapeutic efficacy of surfactants in COVID-19 patients.

The recombinant fragment of human lung surfactant protein D (rfhSP-D) is reported to be more potent than remdesivir, an antiviral, in inhibiting the replication and infectivity of SARS-CoV-2 and the activity is found to mediated through down regulation of RdRp gene expression (Hsieh et al., 2021; Madan et al., 2021).

Computational fluid dynamics simulation studies (Kitaoka et al., 2021) using 3D human airway models has predicted that wedge instillation of pulmonary surfactant from subsegmental bronchi is better than conventional method to deliver the effective concentration of pulmonary surfactant to the lungs to protect them from COVID-19 infection.

Hideyuki has put forward a hypothesis (Takano, 2020) based on cumulative scientific evidences that pulmonary surfactants or synthetic surfactants or surfactant production stimulants may be effective for either prophylaxis or treatment for COVID-19. However, this hypothesis is yet to be tested and validated in clinic.

## Clinical Trials on Pulmonary Surfactants in COVID-19 Patients

Based on the data retrieved from https://clinicaltrials.gov/, accessed on June 15, 2021, the details of on-going clinical trials in surfactants related to COVID-19 are provided in Table 3. Three surfactant products: poractant alfa, bovine lung extract surfactant (BLSE), and lucinactant are in phase I/II trials to test their efficacy in improving the clinical outcomes of ARDS in COVID-19 patients. There are two trials that are underway on poractant alfa using two different routes of administration: bronchial fibroscopy and endotracheal intubation. Another two trials are underway on BLSE using two different routes of administration: endotracheal intubation and inhalation. As for lucinactant, one trial is underway, and it is administered via injection only. In addition, two trials are going on to determine the levels of surfactants present in the lungs and serum of COVID-19 patients with the objective of finding the association between the surfactant levels and ARDS symptoms. Lastly, one clinical trial is underway to determine the efficacy of new drug delivery system directly to the lungs using COVsurf.

**TABLE 3:** List of natural and synthetic surfactant proteins.

Trade name	Surfactant	Type
Curosurf®	Porcine surfactant	Natural
Survanta®	Modified version of bovine surfactant	Natural
ALEC®	Combination of DPPC and egg phosphatidylglycerol	Synthetic
Exosurf®	Combination of DPPC with hexadecanol and tyloxapol	Synthetic
Surfaxin®	SP-B analog KL4	Synthetic
Venticute®	Recombinant human surfactant protein C	Synthetic

## Conclusion

SARS-CoV-2 uses ACE-2 receptor on lungs for entry and alveolar type II cells for replication. Infection with SARS-CoV-2 causes ARDS which may lead to respiratory failure. AT II cells are the sites of pulmonary surfactant production. Lack of PS is the principal cause for NRDS and viral infections are known to reduce PS levels in lungs. PS therapy is the mainstay for NRDS treatment across the world for many years. The results from clinical trials on the efficacy and safety PS in adults with ARDS were not significant in terms of clinical outcomes but they were proven to be safe. The lack of efficacy is attributed to the insufficient delivery of PS to the lungs and thus research has been initiated to investigate new drug delivery systems for improving the PS delivery directly to the lungs. Serum PS levels were found to be low in COVID-19 patients and ARDS clinical symptoms in COVID-19 were found to be like those of NRDS. The science of pulmonary surfactant has come a long way since it was discovered in the 1950s and provides very strong theoretical evidence suggesting that PS could play a role in COVID-19 treatment. In the current COVID-19 pandemic crisis, researchers and health care workers across the globe have been working hard to find a solution to end the pandemic. Few clinical trials are in progress to test the efficacy of three pulmonary surfactants in improving the clinical outcomes in COVID-19 patients, to determine the association between surfactant levels and severity of ARDS in COVID-19 patients, and new drug delivery systems for improved and safe delivery of PS in COVID-19 patients.

## Abbreviations

AA, amino acid; ACE-2, angiotensin converting enzyme 2; ARDS, acute respiratory distress syndrome; AT-II, alveolar type-II; COVID-19, Coronavirus disease 2019; CPAP, continuous positive airway pressure; DAD, diffuse alveolar disease; DPPC, dipalmitoyl phosphatidylcholine; HIV, human immunodeficiency virus; NCT, National Clinical Trials; NL, neutral lipid; NRDS, neonatal respiratory distress syndrome; PC, phosphatidylcholine; PE, phosphatidylethanolamine; PG, phosphatidylglycerol; PL, phospholipid; POPG, palmitoyloleoyl phosphatidylglycerol; PS, pulmonary surfactant; RDS, respiratory distress syndrome; RSV, respiratory syncytial virus; SARS-CoV-2, Severe acute respiratory syndrome coronavirus 2; SP, surfactant protein.
